# Chronic Disease Among Medicaid Beneficiaries Over 50: The Potential Impact of Medicaid Work Requirements

**DOI:** 10.1093/geroni/igab046.1395

**Published:** 2021-12-17

**Authors:** Rodlescia Sneed, Graham Gardner, Alexander Stubblefield, Briana Mezuk

**Affiliations:** 1 Michigan State University, Flint, Michigan, United States; 2 Michigan State University, Michigan State University, Michigan, United States; 3 Michigan State University, East Lansing, Michigan, United States; 4 University of Michigan, Ann Arbor, Michigan, United States

## Abstract

Since 2018, several states have proposed requiring work or other community engagement activities as a condition of receiving Medicaid; however, there has been little inquiry into the impact of such policies on Medicaid recipients over 50. Here, we describe the prevalence and burden of chronic disease among Medicaid beneficiaries over 50 who might be impacted by Medicaid work requirements. We used data from the 2016 wave of the Health and Retirement Study, a large-scale population-based study of adults aged >50. Our sample included individuals over 50 who were not Medicare-eligible (<65 years old) and not receiving Social Security Income. We used logistic regression models to compare those working <20 hours per week (the minimal community engagement/work cutoff) to those working >=20 hours per week, adjusting for age, race/ethnicity, sex, education, and marital status. Individuals working <20 hours per week had greater prevalence of chronic health conditions, including greater odds of diabetes (OR: 2.06; 95% CI: 1.37-2.46), hypertension (OR:2.56; 95% CI: 1.69-3.89), arthritis (OR: 2.96; 95% CI: 2.06-4.62, and lung disease (OR: 4.11; 95% CI: 2.31-7.32). Further, among those with chronic health conditions, those working <20 hours per week reported more, medication use, more worsening of their conditions in the past 2 years, and more hospitalization than their counterparts. Taken together, these finding suggest that Medicaid work requirements in this population would have the most impact on the most medically vulnerable individuals. Policymakers should consider this as they propose policies impacting Medicaid coverage in this population.

